# Equine Cervical Pain and Dysfunction: Pathology, Diagnosis and Treatment

**DOI:** 10.3390/ani11020422

**Published:** 2021-02-06

**Authors:** Melinda R. Story, Kevin K. Haussler, Yvette S. Nout-Lomas, Tawfik A. Aboellail, Christopher E. Kawcak, Myra F. Barrett, David D. Frisbie, C. Wayne McIlwraith

**Affiliations:** 1 Orthopaedic Research Center, Department of Clinical Sciences, College of Veterinary Medicine and Biomedical Science, Colorado State University, Fort Collins, CO 80523, USA; Kevin.Haussler@ColoState.EDU (K.K.H.); Yvette.Nout-Lomas@colostate.edu (Y.S.N.-L.); Christopher.Kawcak@ColoState.EDU (C.E.K.); Myra.Barrett@colostate.edu (M.F.B.); David.Frisbie@ColoState.EDU (D.D.F.); Wayne.Mcilwraith@ColoState.EDU (C.W.M.); 2 Department of Microbiology, Immunology, and Pathology, College of Veterinary Medicine and Biomedical Science, Veterinary Diagnostic Laboratory (VDL), Colorado State University, Fort Collins, CO 80523, USA; Tawfik.Aboellail@ColoState.EDU; 3 Department of Environmental and Radiological Health Sciences, College of Veterinary Medicine and Biomedical Science, Colorado State University, Fort Collins, CO 80523, USA

**Keywords:** horse, neck, myofascial examination, hyperesthesia, conflict behavior, poor performance

## Abstract

**Simple Summary:**

Neck pain and dysfunction in the horse is becoming an increasingly important topic among riders, trainers and veterinarians. Some horses may present for a subtle performance decline, while others may show dramatic, dangerous behavior. It is important to recognize how to carefully evaluate the horse in an effort to understand the different types of pain that may be contributing to the different behaviors. The musculoskeletal and nervous systems may both play a role in the development of clinical signs. Recognizing that there are many diagnostic options as well as several treatments choices is important. This synopsis covers the disease processes that may contribute to the development of neck pain and dysfunction in the horse, as well as several possible diagnostic and treatment options.

**Abstract:**

Interest in the cervical spine as a cause of pain or dysfunction is increasingly becoming the focus of many equine practitioners. Many affected horses are presented for poor performance, while others will present with dramatic, sometimes dangerous behavior. Understanding and distinguishing the different types of neck pain is a starting point to comprehending how the clinical presentations can vary so greatly. There are many steps needed to systematically evaluate the various tissues of the cervical spine to determine which components are contributing to cervical pain and dysfunction. Osseous structures, soft tissues and the central and the peripheral nervous system may all play a role in these various clinical presentations. After completing the clinical evaluation, several imaging modalities may be implemented to help determine the underlying pathologic processes. There are multiple treatment options available and each must be carefully chosen for an individual horse. Provided is a synopsis of the current knowledge as to different disease processes that can result in cervical pain and dysfunction, diagnostic approaches and treatment strategies. Improving the knowledge in these areas will ideally help to return horses to a state of well-being that can be maintained over time and through the rigors of their job or athletic endeavors.

## 1. Introduction

It is becoming increasingly recognized that many horses presented to equine practitioners for poor performance have underlying cervical axial skeletal lesions that result in pain syndromes and an inability to meet athletic demands. However, understanding exactly which structures within the cervical region are affected remains difficult and a potential source of frustration. Unfortunately, diagnostic imaging modalities often fail to help to fully elucidate the underlying disease process, which is similar to what is seen in human patients [1]. The prevalence of neck pain in humans ranges from 30% to 50% [2] and appears mostly associated with abnormal joint motion and disc degeneration [3,4,5]. However, human physicians also struggle to identify the source of neck pain even after employing advanced imaging modalities or other diagnostic techniques and obtaining verbal feedback from their patients. This underscores the challenges that we face in equine practice to understand and diagnose this frustrating and potentially debilitating condition in horses. Due to the paucity of peer-reviewed equine literature on this topic, the information discussed here is a hybrid of a literature review, which includes human and other animal species as needed to delineate specific concepts, combined with the authors’ clinical and research experience. The goal is to provide a synopsis of the current knowledge of common disease processes, diagnostic approaches, and treatment strategies used for managing cervical pain and dysfunction in horses. This synopsis is meant to highlight the many topics and considerations when dealing with a horse presenting for concerns related to the neck. As information is being added to the literature at a rapid rate, it is important for veterinarians presented with these types of cases, to stay abreast of new material. It is the authors’ opinion that as more and more practitioners and riders begin to recognize the complexity of these cases, we can work together to ultimately improve the clinical outcome of these challenging cases.

## 2. Pain Mechanisms

The International Association for the Study of Pain defines pain as “an unpleasant sensory and emotional experience associated with actual or potential tissue damage” [6]. General categories include pain of nociceptive, inflammatory, and pathological mechanisms. Pain management strategies or treatments need to be targeted specifically depending on the type of pain present [7]. Nociceptive pain is protective and the immediate response of the body that serves to limit contact with noxious stimuli by reflexive withdrawal in an effort to constrain tissue damage. Nociception is the “neural process of encoding and processing noxious stimuli” [8]. Afferent sensory fibers have cell bodies located within the dorsal root ganglion. Sensory input from the periphery is transmitted through the dorsal nerve root into the dorsal horn of the spinal cord where it synapses with interneurons and is relayed to the sensory cortex via the ascending spinal cord tracts [9]. Inflammatory pain is characterized by hypersensitivity of injured, inflamed tissues caused by stimulation of the local immune system. Inflammatory pain is also protective in that it limits joint or soft tissue movement or contact with the affected area allowing healing. In contrast, pathologic pain serves no biologic advantage [7] and often induces chronic or maladaptive pain that persists well beyond the presence of the initiating stimulus [10]. Pathological pain is categorized as neuropathic when there is direct injury to the nervous tissues, or dysfunctional when there are no organic lesions of the nervous tissues [7]. Neuropathic pain has been described in horses with laminitis [11], trigeminal-mediated headshaking [12], and has been proposed as a component of the pain associated with osteoarthritis (OA) [13]. It is possible that horses demonstrating cervical pain could be experiencing any of these types of pain, or possibly a combination of pain types.

## 3. Cervical Dysfunction

Dysfunction simply implies impaired or abnormal functioning [14]. Clinical signs of cervical dysfunction in human patients include decreased range of motion, altered body awareness and muscle activity [15], cervical dysfunction has been described in equine patients with subtle gait abnormalities and abnormal muscle tone [16]. In horses presented for poor performance, dysfunction is a critical, yet infrequently used term. Signs of cervical dysfunction may include regional or generalized muscle asymmetry (e.g., atrophy, hypertrophy, and hypertonicity), stiffness or inability to move the neck through a normal range of motion, and altered head or neck carriage [16]. Cervical dysfunction may contribute to altered gait and biomechanics of the forelimb and trunk, producing additional dysfunction, pain and lameness.

## 4. Manifestations of Cervical Pain and Dysfunction

### 4.1. Clinical Presentation

There is a wide spectrum of clinical signs associated with cervical pain and dysfunction. Horses with cervical pain display obvious discomfort associated with palpation or active neck movements in work, as well as during stretching exercises or even daily routines. In contrast, horses that have cervical dysfunction, without overt pain, may display more subtle signs of avoidance, it is possible for horses to display combined signs of pain and dysfunction. Affected horses in either category may have a history of a general decline in performance, neck pain and stiffness, an unwillingness to work on the bit, subtle hind limb gait abnormalities and lack of impulsion [17] and possibly forelimb lameness [18].

Horses with cervical dysfunction are often simply stiff or unwilling to be soft in the bend of their neck and body, may have difficulty with performing certain movements such as smaller circles, or they may pull against the reins or start tossing their head. While some horses are presented for a decline in performance or resisting work, other horses are more dramatic in their presentation. These horses may stop and refuse to go forward and may even rear and flip over backwards if the rider continues to ask in more forceful ways. Cervical radiculopathy in humans is a neurologic disorder that results from nerve root dysfunction either from mechanical compression or from local inflammatory mediators, and may result in myelopathy and muscle weakness [19]. In horses, cervical radiculopathy typically results in localized pain within caudal cervical region and forelimb lameness due to peripheral nerve contributions from the brachial plexus [20]. An unexplained change in behavior is another common clinical sign recognized in horses with cervical pain or dysfunction. These horses display sudden onset of spooking within familiar surroundings or they are reported to act fearful [21]. Riders and trainers may not always recognize these subtle behavioral changes, which may only be identified while acquiring a detailed history or be seen during on-site or ridden examinations. It is also possible for affected horses to develop apparent hypersensitivity whereby they resist being saddled, brushed, or even touched. Sometimes these horses even avoid the typical social greeting at the stall door or being caught. Occasionally, horses are presented for concerns that seem unrelated to cervical pain, such as weight loss seen in horses with cervical pain that precludes them from reaching food on the ground or requires twisting their head to eat from a feeder. With many different clinical presentations, the practitioner must use detailed observation and all other forms of clinical information available to arrive at a diagnosis of neck pain and dysfunction.

### 4.2. Observation

When evaluating horses with neck pain and dysfunction, clinicians should perform a detailed, multistep examination and be careful not to overlook any perceived subtleties. Observing the natural behavior of horses in their home environment is helpful as the stress and excitement of being in unfamiliar surroundings will cause some horses to override signs of pain and discomfort. Behavioral assessment is an important part of the overall examination to take note of [22]. Careful attention should be paid to the horse’s stance, facial expressions, and how the horse interacts with its surroundings and humans. The established horse grimace scale is helpful in assessing signs of pain through changes in facial expressions, such as subtle eye, ear, or mouth positions and characteristics [23]. Assessing neck posture at rest relative to the position of head, limbs and trunk may help identify abnormalities [17]; for example, horses with caudal cervical pain may shift their weight or alternate their forelimb placement from a normal square stance to a position with one limb predominately protracted and the other retracted. When encouraged to come to the stall door, horses may keep their necks in a very extended and rigid posture and only move their eyes to look toward the handler. Observing regional and left-right differences in muscle symmetry and development can help identify areas of muscle atrophy or hypertrophy that may indicate underlying disuse or neurogenic muscle atrophy [24]. Abnormal sweat patterns along the lateral cervical region may also indicate signs of sympathetic dysfunction [25].

### 4.3. Digital Palpation

Systematic and detailed palpation of the soft tissues and bony landmarks within the cervical region is a critical step in identifying and localizing potential sources of neck pain and dysfunction (e.g., stiffness, muscle hypertonicity) (Supplemental material: Video S1). Soft tissues should be palpated from superficial to deep with specific focus on assessing texture and tone changes within the different tissue layers. Starting at the poll and continuing along the dorsal, lateral and ventral regions of the neck, the quantity (e.g., size or area) and the quality (e.g., severity) of heat or swelling is noted, which may be indicative of inflammation. The fascia is examined for tone and signs of tightness or reactions to the initial movement of the skin. The skin should glide freely in craniocaudal and dorsoventral directions over the underlying muscles without any signs of resistance or pain. Muscle tone and symmetry, and myofascial trigger points, which are discrete, painful, taut bands within the muscle that generate a referred pain pattern, are evaluated [26]. Muscle development is assessed by examining the surface contours of the lateral cervical region and identifying regions of convexity (i.e., well-developed), flat (i.e., lack of development), or concavity (i.e., muscle atrophy). A common site of muscle atrophy or lack of development can be identified within the lower cervical region (C4–C6) affecting the splenius and semispinalis muscles. The brachiocephalic muscles are commonly painful on manual compression in horses with lower cervical pain or stiffness, which can be identified with firm compression of the muscle beginning at the upper and progressing to the lower cervical region. Finally, an avoidance response after deep palpation over the cervical transverse processes and articular processes might indicate the presence of bone or joint pain.

### 4.4. Dynamic Spinal Examination

For the dynamic spinal examination, both passive and active spinal movements are analyzed. Passive spinal movements are applied to assess joint and soft tissue movement without muscle activation, whereas active spinal movements consist of the patient initiating the motion, which requires muscle activation [27]. In order to be as specific as possible with respect to the individual vertebrae affected in normal or dysfunctional cervical motion, active exercises are performed with the horse placed against a wall or in a corner, so it does not step away when being asked to perform the specified movements [17]. It is important to evaluate lateral bending and the ability to flex and extend the entire head and neck region from both quantitative (e.g., range of motion) and qualitative (e.g., ease and fluidity) perspectives. Some horses resist some or all of these movements, which may relate to pain stemming from individual articulations or due to overall stiffness or muscle guarding throughout the entire neck. Treats may be used to encourage neck movement for active mobilization; however, some horses are not well motivated by food, while others may aggressively lunge for the treats. Horses with normal neck mobility can readily move their heads and necks from side to side and position their chin near the girth, hip, or tarsus [28]. When asking the horse to go through these lateral bending movements, most of the mobility occurs within the cranial and caudal cervical regions, with less mobility noted at C2–C5 [28]. Some horses will quickly rotate their heads axially at the atlantoaxial articulation when asked for lateral bending and are unable to bend in the middle or lower cervical regions due to stiffness or pain. If a horse is painful and unable to readily laterally bend its neck, then repeating the motion while keeping the chin further away from the body by approximately 30 cm may be helpful [28]. Evaluating flexion and extension is used to assess mobility at the atlantooccipital articulation and lower cervical region. These exercises include asking the horses to extend their nose out in front of them and elevating it as high as possible. Flexion exercises typically include movements along the mid-sagittal plane where the nose is brought toward the point of chest, carpus and fetlock region [29].

### 4.5. Gait Examination

Horses with cervical pain and dysfunction should undergo a thorough evaluation of gait, including assessment for lameness and neurologic disease. The axial skeleton can play an important role producing subtle gait abnormalities, which might be initially missed by riders, trainers, and veterinarians who are focused on issues affecting the appendicular skeleton. Routine lameness examination is aimed at identifying gait abnormalities stemming from appendicular musculoskeletal disease; however, it is important to recognize that careful static and dynamic examination of the axial skeleton is necessary to assess axial-appendicular interactions and possible compensatory gait mechanisms. In addition to the routine lameness examination performed at the trot on a straight line and circles, it is beneficial to examine affected horses under tack at different gaits. Abnormalities in head or neck carriage may only be appreciated on the lunge line with the head carried toward the outside of the circle (i.e., difficult to turn in one direction) or gait deficits noticed with or without using side reins. Other horses may have pronounced lowering of the head and extension through the lower cervical region and appear to have generalized neck stiffness. Horses may compensate for cervical pain or dysfunction by changing forelimb flight patterns (e.g., hopping-like motion) or by asymmetrical gait patterns due to cervical radiculopathy [20,30]. Some affected horses appear to have an apparent weakness or difficulty in fully engaging the forelimbs in the early stance phase, which may precipitate stumbling. Forelimb lameness that cannot be localized to the limb with diagnostic analgesia may originate from the cervical region. Intra-articular (IA) analgesia of the articular process joints (APJ) can be considered in select cases to help confirm cervical localization of pain and inflammation as the source of lameness [20,30,31]. Horses that are generally stiff and resistant, however, do not seem to be good candidates for IA analgesia of the APJs as it is often difficult to gauge a level of improvement due to the high likelihood of multiple vertebral levels being affected.

A complete neurologic examination should also be performed to determine whether neurologic disease could be contributing to the observed clinical signs. Specifically, diseases such as cervical vertebral compressive myelopathy (CVCM), equine degenerative myelopathy, and equine protozoal myeloencephalitis should be considered. Ancillary diagnostics such as cervical myelography and serum and CSF SAG2,4/3 antibody testing can be pursued, as indicated [32]. Further, the presence of underlying myopathies including polysaccharide storage myopathy, vitamin E-related myopathies, and myofibrillar myopathies should be considered. An exercise challenge with evaluation of muscle enzymes and muscle biopsies can be pursued if indicated [33].

## 5. Osseous Sources of Cervical Pain and Dysfunction

### 5.1. Cervical Articular Process Joint

Synovitis and associated joint pain are commonly diagnosed as a cause of poor performance in horses [17,18]. The APJs contribute to the spinal motion unit, which consists of the two dorsally-paired diarthrodial articulations and the ventral intervertebral disc. In horses, two commonly noted abnormalities affecting the APJs are osteochondrosis (OC) and OA. Osteochondrotic lesions vary from small fissures within the articular surface to large irregularities with evidence of secondary OA [34]. OC diagnosed via imaging and histopathology has been noted in young horses with CVCM [35]. Horses with OC of the cervical articular surfaces, as is true of the appendicular skeleton, may have subtle to no clinical signs associated with the lesions. Other horses, however, may experience progressive inflammation and pain of the APJ secondary to OC [36].

OA is a disease of the cartilage surface and bone structure; however, it is important to recognize that other structures within the joint complex, which include subchondral bone, joint capsule, synovium, and paraspinal muscles, are also affected and can be a primary source of cervical pain [37]. Radiographic evidence of OA of the APJ includes joint enlargement, subchondral sclerosis, extension of the dorsal laminae, joint margin lipping, and the presence of osteophytes and joint capsule enthesophytes [38]. A recent post-mortem study in a mixed population of horses showed that the most commonly noted bony changes on the caudal articular processes were modeling and joint margin flattening, while the most common changes noted on the cranial articular processes were osteophyte formation, joint margin lipping and enthesophytes of the joint capsule with the most severe changes noted at C2–C3 and at C7–T1 [37]. Interestingly, a retrospective study found that the C5–C6 APJs are enlarged with no correlation to clinical signs [39] and it has been reported that approximately 50% of normal mature horses have some degree of unilateral or bilateral changes in cervical joint margins [39]. These findings make it impossible to diagnose cervical pain or dysfunction solely based on radiographic images and caution should be used to avoid the overinterpretation of OA as the primary cause of the observed clinical signs.

### 5.2. Vertebral Body

Morphologic variations affecting the vertebrae have been documented extending from the occiput [40] to the cranial thoracic region [41]. Of particular interest in performance horses are malformations affecting the caudal cervical vertebrae, which may include unilateral or bilateral transposition of the ventral process from C6, variation in the size and shape of the C7 spinous processes, and the presence of additional ribs or costal processes [41]. Improvements in diagnostic imaging techniques provide better visualization of these areas [42,43], which will ultimately allow for an increased understanding of the clinical significance of these malformations. While the clinical significance has been questioned [44], horses with transitional anomalies of the C6 lamina have been reported to have an increase in perceived cervical pain and decreased joint range of motion, likely secondary to altered attachment of the longus thoracis muscle [45].

While fractures of the cervical vertebrae usually follow acute trauma [17], affected horses may also present with neck stiffness and poor performance without any known trauma. As indicated in any suspected fracture, radiography is a first-line imaging tool to confirm the diagnosis. In some cases, the fracture may not be easily identified and computed tomography (CT) may be indicated. Judicious use of non-steroidal anti-inflammatory drugs (NSAIDs) is indicated in acute neck pain cases along with confinement and management strategies such as offering food and water at a neutral head and neck level, which may help to minimize induced motion at the fracture site [17]. As healing progresses, it is important to monitor the comfort of the horse, amount of callous formation, and possible consequences to the APJs (e.g., osteoarthritis) and vertebral canal (e.g., CVCM). Moreover, it is possible for the vertebral segments cranial and caudal to the fracture site to be impacted by altered biomechanics of the neck. Prognosis is influenced by the structures involved in the fracture and healing process, the degree of instability at the fracture site, and the neurologic status of the horse.

## 6. Soft Tissue Sources of Cervical Pain and Dysfunction

### 6.1. Cervical Fascia

The fascia is connective tissue that surrounds and connects every muscle and organ, forming a continuous collagen network within the body. The superficial fascia is highly vascularized and innervated, while the deep fascia has a role in isolating individual muscles or muscle groups and providing attachment for muscles [46]. In humans, the deep fascia has been found to be the most pain-sensitive tissue in the thoracolumbar region in both acute and chronic pain conditions [47]. The deep fascia can undergo densification and fibrosis which changes the ability of the tissues to glide and alters the nerve fiber function, leading to an increased pain state [48]. The fascia specific to the horse has recently been evaluated and found to be quite compact and tightly connected, likely related to the need for an energy-efficient system [49]. In horses with cervical pain and dysfunction, compensatory nociceptive and biomechanical mechanisms likely contribute to densification of the fascial tissues and the development or chronic recurrence of pain.

### 6.2. Cervical Muscles

The cervical musculature is likely an important source of neck pain and dysfunction. The brachiocephalicus muscle is a superficial muscle that extends from the occipital bone to the humerus, while the adjacent omotransversarius muscle extends from the wing of the atlas and inserts on the cervical transverse processes and on the spine of the scapula. Both of these muscles serve a primary role in shoulder extension and forelimb protraction [50]. If these muscles are painful or weak, horses may present with a decreased cranial phase of the stride and reluctance to laterally bend away from the affected muscle. The splenius muscle contributes to lateral bending of the head, prevents unwanted flexion of the head during movement, and provides static postural support [51]. The dorsal portion of the semispinalis muscle imparts passive support to the head and neck; while the ventral region plays a more active role to raise the head [51]. The splenius and semispinalis capitis muscles and nuchal ligament all function to resist gravitational forces and to actively elevate the head and extend the neck [52]. The deep cervical musculature function in stabilizing individual cervical intervertebral joints and consist of dorsal, lateral and ventral muscle groups, which include the multifidus, intertransversarius and longus colli muscles, respectively [37,52]. The multifidus cervicis is a complex muscle with multiple intersegmental fascicles from the level of C2 through the cervicothoracic junction [52]. The fascicles of the multifidus have caudal attachments to the joint capsules of the APJ [53] and proper function of these stabilizing muscles is important for neuromotor control, proprioception and joint stability. In humans, atrophy or weakness of the multifidus and longus colli muscles are often associated with whiplash injuries and neck pain [54]. Segmental atrophy of the multifidus muscle has also been observed in horses associated with APJ pathology [52,55]. The longus colli muscle also has distinct intersegmental fascicles from C1 through C5. At the level of C6–T5, this multifascicular structure is replaced by a single muscle belly to form the longus thoracis muscle which attaches to the caudal C6 transverse [52]. Variations of attachment sites of the longus colli, secondary to developmental anomalies of C6, have been shown to be associated with proprioceptive and neurologic dysfunction [56]. This highlights the importance of anatomical features, articular pathology, and functional control of the cervical muscles to prevent or limit the development of neck pain and dysfunction.

### 6.3. Nuchal Ligament Desmopathy

The nuchal ligament is a bilobed, highly elastic structure that connects the occiput and cervical vertebra to the cranial thoracic spinous processes. Occipital enthesophytes have been reported in 85% of 302 warmblood horses aged 1–22 years [17]. Horses with suspicion of nuchal ligament enthesopathy or desmopathy may be unwilling to position their head straight in the bridle, resist poll flexion, and may have inconsistent responses to soft tissue palpation [17]. Radiography and ultrasonography are indicated to evaluate the attachment sites of the semispinalis tendon along the caudal occiput and the fiber pattern and attachment of the nuchal ligament. Local anesthetic infiltration may be warranted to determine the clinical significance of positive radiographic findings and the response to ridden exercise and induced poll flexion. Caution is needed to avoid the occipitoatlantal epidural space during local anesthetic infiltration to avoid inducing ataxia [17]. Treatment trials with acupuncture, spinal mobilization, laser therapy, and extracorporeal shockwave therapy (ESWT) may help to improve the clinical signs associated with enthesopathies in this location. Surprisingly, horses that avoid palpation of the poll region seem to respond well to ESWT and generally require minimal sedation. Using a piece of felt under the crown piece of the bridle, or using a headstall designed to redirect pressure more caudally over the poll region may also reduce the clinical signs in some horses.

### 6.4. Nuchal Bursitis

Bursal inflammation can stem from infectious and non-infectious causes that result in pain, stiffness, and abnormal head and neck postures. The funicular portion of the nuchal ligament travels over the dorsal aspects of the atlas (C1) and axis (C2) as it attaches to the caudal aspect of the occiput. The cranial (dorsal to C1) and caudal (dorsal to C2) nuchal bursae are potential spaces within the fascial layers and are not readily identifiable unless filled with fluid. Localized swelling can sometimes be palpable, but a more definitive diagnosis is possible with ultrasonography [57]. Therapeutic options include medical therapy (i.e., rest, NSAIDs with or without intrabursal treatment), as well as surgical debridement, both may be curative in some horses [58].

### 6.5. Cervical Joint Capsule Fibrosis and Synovitis

Stretching or injury to the articular process joint capsule is considered an important source of cervical pain in humans [9,59] and goats [60]. A high density of A-delta and C fiber receptors within the joint capsule have nociceptive as well as proprioceptive roles [61]. Capsular microdamage is capable of evoking pain through sustained nociceptive firing [62]. As the multifidus muscle attaches directly to the cervical joint capsule in both humans and horses, dysfunction of the multifidus cervicis muscle and subsequent altered cervical biomechanics with increased forces applied to the joint capsule, may play an important role in the etiopathogenesis of equine cervical pain [52]. Synovitis may also be a significant source of neck pain through pressure from increased joint effusion and inflammatory mediators (e.g., metalloproteinases, prostaglandins, free radicals, and a number of cytokines including IL-1β and TNFα) [63].

### 6.6. Cervical Intervertebral Disc Disease

Intervertebral disc disease is common in humans [64] and dogs [65,66]. In horses, there are limited case reports mostly focusing on end-stage disc degeneration and vertebral endplate eburnation [67,68]. More recently, the earlier stages of intervertebral disk degeneration of horses have been reported [69,70]. In contrast to previous literature [71], the study by Bergmann suggests that the intervertebral disc has a grossly and histologically discernible proteoglycan-rich central region that is judged to be different from the lamellar collagenous annulus fibrosus [69]. In this study, they proposed a grading scheme for intervertebral disc degeneration that had very good intra- and interobserver reliability which may be useful for future research. In a previous report of end stage disease [68], horses showed severe neurologic dysfunction including pelvic limb ataxia and recumbency. However, early stages of disc degeneration and segmental instability may produce subtle signs of spinal cord compression in addition to cervical pain and dysfunction and possible lameness [70].

## 7. Nervous System Structures in Neck Pain

### 7.1. Spinal Nerve Roots

Spinal nerve roots exit through the intervertebral foramen (IVF) and have been reported to be at risk of impingement [18] or possible radiculopathy secondary to severe OA of the APJ [72], similar to what is seen in humans. Although a recent publication characterizing bony changes of the APJ in horses [37] did not identify size limitations of the IVF to cause physical impingement, and investigators were not able to create nerve root impingement after distending the APJ capsule [73], there appears to be clinical evidence to suggest nerve root impingement or a similar syndrome occurs in the horse [74]. It is also likely that inflammatory mediators associated with APJ OA contribute to chemical-induced neuritis and the development of neck pain and dysfunction. This may be difficult to confirm in vivo, but histologic evidence of nerve root injury has been noted at post-mortem examination in horses with unexplained forelimb lameness [20]. The in vivo diagnosis of cervical radiculopathy or neuritis is still presumptively based on collective clinical examination findings, radiographs suggestive of APJ arthropathy, electromyography, three-dimensional imaging, and exclusion of other causes of cervical pain and dysfunction. It is the authors’ opinion that these horses are often quite painful and reactive, not just stiff and mildly resistant. Affected horses may be quite explosive and unpredictable in their reaction to being asked to bend and engage their cervical region. As has been described, horses may present for a forelimb lameness that cannot be localized, or with a “hopping-limb” forelimb lameness [20].

### 7.2. Dorsal Root Ganglia

The dorsal root ganglia (DRG) consist of clusters of neuronal cell bodies from the afferent sensory neurons. Their location, at or within the IVF, results in a potential increased risk of injury to the sensitive neuronal tissue. Unlike the central nervous system, there is no neurovascular barrier protecting the DRG. In the horse, ganglionitis has been described related to chronic laminitic pain [11,75] and as a proposed mechanism of headshaking and trigeminal neuralgia [76]. More recently, there has been identification of lymphocytic inflammation within or around the DRG in horses identified with “idiopathic hopping-like forelimb lameness” [30]. The clinical relevance of dorsal root ganglionitis and its relationship to equine chronic neck pain remains to be elucidated.

### 7.3. Spinal Cord

The most common cause of spinal cord disease is cervical spinal cord compression seen in CVCM [35,77,78,79]. While CVCM is a common condition in horses presented with varying degrees of symmetric ataxia, evidence of neck pain in this population of horses is not always present. In fact, young horses with developmental orthopedic lesions causing stenosis of the vertebral canal often have no evidence of neck pain. In contrast, older horses with OA of the APJ may have ataxia and have a stiff and painful neck on examination [80], or are presented for neck pain and are found to have subtle ataxia on clinical examination.

## 8. Diagnostic Imaging

### 8.1. Radiography

Radiographs provide a good baseline screening tool for horses with neck pain or dysfunction; however, it is important to recognize that some cervical lesions are radiographically occult and that some findings, such as enlargement of the APJs, may not be clinically significant [24,39]. Radiographs are indicated in horses with a history of cervical trauma, neck pain or stiffness, decreased performance, gait abnormalities associated with neurologic deficits, or forelimb lameness that is not readily localized to the limb. Lateral–lateral radiographs from the caudal skull to the first thoracic vertebrae are readily acquired in the standing, sedated horse to allow evaluation of bones, including morphology and alignment. Radiographs also provide indirect evidence of soft tissue injury, including the presence of spinal cord compression if there is obvious vertebral canal stenosis. The technique for acquiring oblique images of the cervical vertebrae has been described [81] and can improve detection and better localization of bone pathology [82]. The clinical significance of radiographic abnormalities may require further investigation, such as the response to IA analgesia or treatment trial. All radiographic findings must be interpreted in conjunction with the clinical examination findings.

### 8.2. Ultrasonography

Cervical ultrasonography is frequently used as an adjunct to radiographs to further assess changes in cervical APJ margins, joint capsules, and other soft tissues (e.g., nuchal ligament and bursae). A complete examination of the cervical region extends from the caudal occiput to the last visible articulation, typically C7–T1 [82]. Changes of the APJs that can be identified include periarticular bone remodeling and osteophytes, increased joint fluid, and thickened joint capsules. Mild periarticular osteophytes in the absence of joint effusion or capsular change are more difficult to interpret as the significance of this finding is variable. Joint effusion is rarely found incidentally and suggests an active joint disease or inflammation. Accurate evaluation of the IVF and spinal nerve roots is limited due to the oblique angle of the ultrasound probe relative to the underlying anatomy. Cervical ultrasound is not only used for diagnostic purposes, but also to guide administration of medications or anesthetics into the synovial articulations [83,84,85].

### 8.3. Nuclear Scintigraphy

Nuclear scintigraphy is a commonly used tool for the diagnosis of obscure lameness and poor performance in horses; however, it is a relatively insensitive technique in the cervical spine and may produce false-positive results [17]. The normally larger size of the C6–C7 APJ is associated with greater uptake than adjacent articular processes, which can be overinterpreted as a significant finding. Additionally, enlargement and remodeling of the APJs can result in increased uptake with no clinical significance [86]. False negative results are also possible, particularly in the caudal neck, due to the thick overlying muscle mass and scapular shielding [17]. Negative findings on scintigraphy do not rule out pain originating from the cervical vertebrae, rather merely rules out the presence of increased bone turnover. Appropriate image acquisition requires obtaining both left and right lateral images for accurate lesion localization and to better direct therapy [82]. As with all cervical imaging, results must be correlated with other clinical examination and imaging findings.

### 8.4. Three-Dimensional Diagnostic Imaging

Advanced imaging of the cervical spine includes CT, CT myelography, and magnetic resonance imaging (MRI) which has been used to describe normal cervical anatomy [87]. MRI is a non-invasive method to evaluate the cervical nerve roots, but at this time it is not capable of imaging the caudal cervical spine in most adult horses [20]. With increased attention to cervical disease in the horse, the demand for magnet configurations that allow for examination of the entire cervical region may increase the viability of MRI to be used in ante-mortem assessment of the cervical spine, including the IVF and cervical nerve roots. While MRI is the gold standard for cervical imaging, currently CT is more clinically available for imaging the entire length of the equine cervical region. CT provides excellent bone detail and allows for more comprehensive assessment of osseous changes compared to radiographs. For example, CT allows for determination of orientation of APJ enlargement and identification of medial versus lateral extension of the joint margins onto the spinal cord and spinal nerve roots exiting the IVF. Contrast-enhanced CT widens its diagnostic applicability, and can be utilized in whole body, IA, or intrathecal applications. IA contrast helps define the APJ, especially the articular cartilage surface. CT myelograms are commonly indicated to diagnose CVCM [74] as well as having applications in diagnosing OA of the APJ, fractures, malformations and some soft tissue lesions [88]. However, CT lacks the soft tissue detail to assess neuritis, myositis, and early intervertebral disc degeneration amongst other soft tissue injuries; MRI is the optimal modality to assess such soft tissue changes.

### 8.5. Electrodiagnostic Evaluation

Electromyography is used to directly assess the neurophysiologic status of the motor unit and its individual constituents, including the alpha motor neuron, its axon, the motor endplates and the associated muscle fibers [89,90]. This technique is used to differentiate between neurogenic and myogenic disorders, and can provide insight into severity and distribution of lesions. Determination of nerve conduction velocity can provide further insight into function of peripheral nerves. However, proper data collection and interpretation requires a thorough understanding of neuromuscular physiology and associated technical factors [90]. Possible indications include chronic, poorly localized lameness and neurologic dysfunction from unknown causes [91].

### 8.6. Surgical Evaluation

Epiduroscopy is a technique that has been described that allows direct visual inspection of the dorsal and ventral nerve roots to the level of the 8th cervical nerve [92]. This technique is in early experimental stages and is not routinely used clinically. Needle scope arthroscopy has recently been described [93] which has the potential to allow a more complete evaluation of the APJ in a standing horse. Both of these techniques need further exploration into the potential benefits they may offer to the understanding of cervical pain or dysfunction.

## 9. Treatment Options

### 9.1. Systemic Medications

#### 9.1.1. Non-Steroidal Anti-Inflammatory Drugs (NSAIDs)

NSAIDs are the most frequently used analgesics in horses worldwide [94]. When using NSAIDs for treating cervical pain, drug toxicity, drug doses and competition rules should be considered [95]. NSAIDs may not have the desired efficacy in treatment of some affected horses; it is possible that the anti-inflammatory action of NSAIDs is ineffective in pathologic pain conditions, which have no primary inflammatory component. However, in acute injuries with inflammation, NSAIDs are indicated. Long-term dosing with firocoxib may be helpful in some chronic cases of cervical OA. However, it is the authors’ opinion that the complexity of cervical pain and the possibility of a neuropathic pain component makes this medication less rewarding than may be noted when used for appendicular skeleton lameness.

#### 9.1.2. Bisphosphonates

Bisphosphonates have reported effects on bone turnover and therefore may have therapeutic effects to alter the remodeling phase in certain types of bone pathology. Clodronate and tiludronate have been approved in the USA for treatment of navicular disease in the horse [96]. Tiludronate has been reported to improve flexibility in horses with clinical and radiographic findings suggestive of OA of the APJ within the thoracolumbar region [97]. In addition to bone-sparing properties of bisphosphonates, anti-inflammatory effects occur via decreased nitric oxide and cytokines released from activated macrophages [98]. These mechanisms of action make this class of drug a reasonable consideration in horses that present with neck pain and radiographic evidence of bone remodeling when improvement of clinical signs of cervical pain has not been achieved in more commonly used therapies.

#### 9.1.3. Gabapentin

Gabapentin is an antiepileptic agent that is commonly used to treat neuropathic pain in veterinary patients [99] and as a first line treatment in humans [100]. Gabapentin acts on the voltage-gated calcium channels localized primarily at the synapses [101], decreasing neuronal excitability through binding of the α_2_δ-1 ligand and altering pain processing [99]. In rats, gabapentin in combination with either diclofenac or celecoxib has higher efficacy and safety than either drug alone [102]. There are no studies in horses evaluating this synergy, so it is unknown if combination therapy with NSAIDs is necessary for improved efficacy. Gabapentin was not associated with any negative cardiovascular or behavioral effects but was shown to have a low bioavailability of 16% with oral dosing at 20 mg/kg in horses [103]. Gabapentin may be considered as a reasonable treatment in horses with neuropathic or chronic neck pain. Clinically, this is a medication to consider in very painful or hypersensitive horses.

#### 9.1.4. Muscle Relaxants

Muscle relaxants may be used alone or in combination with other medications or modalities such as ESWT when the horse is experiencing hypertonic or painful cervical muscles. While the author does not use muscle relaxants routinely as a first-round treatment, these drugs could be considered for certain cases, such as a horse with a very tight, reactive brachiocephalicus muscle. These horses may have a shortened cranial phase of the stride and be unable to retract the limb comfortably. The author has found that these medications can bring some relief in such instances. Methocarbamol is a centrally acting skeletal muscle relaxant, labelled to reduce muscular spasms [104], and is commonly used as a first-choice drug for these cases. There is a very large dose range for intravenous use of 4.4–55.0 mg/kg. There is not an FDA-approved label dose for oral use; however, it has been reported to be used at 2–3 times the IV dose [105]. Dantrolene, another skeletal muscle relaxant used in horses, acts by suppressing calcium release, subsequently interfering with excitation-contraction coupling in the muscle fiber [106]. There is no FDA-labelled product for use in horses, although the human product may be used off-label, and a dose of 4 mg/kg has been published and shown to decrease serum CK after exercise [107].

### 9.2. Physical Medicine

#### 9.2.1. Chiropractic

Chiropractic in horses is more and more commonly implemented for back pain [108] and there is evidence to support the beneficial effects [109]. While the same has not been reported for equine cervical pain, the physiology of chiropractic medicine supports implementing this therapy for cervical pain and dysfunction. Chiropractic, a form of manual therapy, uses high-velocity, low-amplitude thrusts, specifically aimed at the joint [108]. Chiropractic medicine may be implemented as both a diagnostic or therapeutic modality, with the goal of treatment being to restore normal joint motion, reducing pain, and stimulating neurologic reflexes [110]. Hypomobility of a joint may give rise to a number of problems, including muscle spasm, nerve dysfunction, and pain [111]. Horses with chronic neck pain or dysfunction often show compensatory changes in their biomechanics, which subsequently predisposes them to further injury [108]. Spinal mobilization is indicated for neck or back pain, localized or regional joint stiffness, and commonly for poor performance or asymmetric gait without overt lameness [27]. In human patients, chiropractic is commonly used for management of neck pain. Additionally, chiropractic in humans has been shown to improve proprioceptive input from the cervical spine, which could affect postural control as well as decreasing pain [112]. Contraindications of chiropractic include fractures, neoplasia, spinal instability and acute conditions that require more conventional therapy. Therefore, having a thorough understanding of any underlying pathology or disease state is of utmost importance when implementing chiropractic medicine in horses with neck pain and dysfunction.

#### 9.2.2. Therapeutic Exercise

While performing dynamic exercises of the cervical spine, employing concentric muscle activation, the horse’s posture is altered. Stability of the body and limbs is then achieved through activation of the abdominal, epaxial, and pelvic muscles, through isometric or eccentric actions [29]. Performed in the same fashion as was described in the dynamic spinal examination section, the mobilization exercises aimed at the cervical spine not only mobilize the joints in the cervical region, but may also activate and strengthen both the epaxial and hypaxial muscles along the entire axial skeleton. This whole-body activation and strengthening may change the functional movement patters and neuromotor control [29]. Additionally, when performing dynamic exercises, the range of motion is controlled by the horse which decreases the risk of the joint moving out of the comfort zone, as is the case in passive stretching. There is a more normal neuromotor control when stimulating the muscles that move and stabilize a specific joint [29]. Exercise therapies are critical for the effective management of human patients with neck pain [113]. In humans, the longus colli and longus capitis muscles (i.e., deep cervical flexors) have an important role in joint support and control, which cannot be reproduced by the more superficial muscles [114]. In horses, the multifidus cervicis and longus colli muscles also function to provide dynamic segmental stability and support in the cervical spine [52]. When developing a rehabilitation program to improve joint stability, focusing on these deep paravertebral muscles is an important consideration. As with all rehabilitation protocols, it is important to have a working knowledge of the related anatomy and what tissues are believed to be compromised so that a program can be tailored specifically on a horse-by-horse basis.

#### 9.2.3. Acupuncture

Acupuncture is used to stimulate nerves, muscles, and connective tissues throughout the body with the goal of alleviating pain [115]. As a general concept, it is believed that the insertion of the needle into the skin and manipulation of the tissue with manual acupuncture, or stimulation with electrical currents with electrical acupuncture causes a number of reactions locally, as well as at the level of the spine, and in the brain [116]. Altering the neural activity in response to acupuncture causes the synthesis and release of neuromodulators that have the potential to have a therapeutic effect in many disease states [117]. Neuromodulation with acupuncture is used to control pain and inflammation in humans [116], and likely has a similar physiologic response in horses. In rodent models, it has been shown that acupuncture may protect against articular cartilage erosion [118] as well as chondrocyte inflammation [119], and these effects may help in clinical cases of cervical OA. It is the authors’ opinion that many horses experiencing neck pain respond well to acupuncture of the cervical region and acupuncture may help prolong treatment intervals when used in conjunction with other therapies.

#### 9.2.4. Mesotherapy

Mesotherapy is a minimally invasive technique where small doses of medications are given intradermally in regions of musculoskeletal pain. Mesotherapy is used for many conditions in human medicine, with pain management being a primary justification [120]. Common medications used for injection include local anesthetics, corticosteroids or saline in order to disrupt the local pain reflex arc [55]. In equine practice, mesotherapy is thought to be useful in the treatment of neck pain, reducing muscle spasm and improving the range of motion in horses with chronic neck pain [55]. For practitioners trained in acupuncture, mesotherapy is likely less frequently utilized, as some of the pain inhibition pathways are quite similar.

#### 9.2.5. Electrotherapy

There are multiple forms of electrotherapy that may be used for pain management. Some of the more common modalities utilized include transcutaneous nerve stimulation (TENS), pulsed electromagnetic field therapy (PEMF), and neuromuscular electrical stimulation (NMES). It is believed that TENS therapy modulates pain through activation of the descending inhibitory system as well as by increased release of endogenous opioids [121]. Although there is limited research to support using TENS therapy for pain relief in horses [122], there are data on humans with knee OA to show a beneficial effect on knee pain [123]. PEMF therapy uses electromagnetic fields to produce secondary electrical currents in a tissue and has been used to provide pain relief as well as improving function in humans with OA [124]. In a study in ponies with induced carpal synovitis, PEMF treatment showed a positive effect [125]. There are multiple ways to employ PEMF including coils, blankets and wraps [122]. NMES may be used for muscle development and stimulating neuromuscular control [126] by causing depolarization of a motor neuron. A specific type of NMES used in horses is a functional electrical stimulation (FES) unit [122], which has been shown to improve functional movement and decrease epaxial muscle spasm in horses [127]. While this is encouraging, the literature in humans is inconsistent, and some horses may be apprehensive of this type of therapy [126].

#### 9.2.6. Extracorporeal Shockwave Therapy

The mechanism of action of ESWT is not fully understood; however, there is good evidence of an immediate effect of this technique on pain receptor physiology as well as initiation of fascial tissue healing [128]. The use of ESWT for horses was initially adapted from human medicine where a positive effect for treating insertional desmopathies was found [129]. ESWT is commonly utilized for treatment of horses with nuchal ligament desmopathy with reported positive results [129]. ESWT has also been shown to decrease pain and improve cervical range of motion in human patients with myofascial pain syndrome [130]. ESWT has recently been shown to raise the mechanical nociceptive threshold in horses with back pain [131]. Anecdotal evidence suggests that horses with myofascial pain and restricted mobility of the cervical spine also respond favorably to ESWT. In addition to the soft tissues that may respond favorably, ESWT has been shown to be beneficial in the treatment of osteoarthritic conditions in human and veterinary medicine [132,133] and therefore may also help horses with cervical OA.

#### 9.2.7. Elastic Therapeutic Tape

Elastic therapeutic tape is used to increase local circulation and therefore reduce oedema, give stimulation to the skin, muscle or fascia and provide afferent input to the central nervous system [134]. In human medicine, there are limited data to support short-term pain relief and cervical range of motion with elastic therapeutic tape [135]. In equine practice, the use of elastic therapeutic tape has become quite common, with applications from the competition ground to rehabilitation facilities [136]. While the use of therapeutic taping is growing, it is important to recognize the mechanisms of action and possible beneficial outcomes remain unclear. While there is some thought that tape can have pain-relieving effects, therapeutic taping is more often used for equine cervical dysfunction.

### 9.3. Local Therapies

#### 9.3.1. Intra-Articular Corticosteroids

While many horses will respond positively to less invasive management strategies, horses with clinical signs and imaging findings consistent with cervical OA frequently benefit from IA application of corticosteroids, which is commonly performed in horses with cervical pain [137]. Birmingham reported that 71% of symptomatic horses returned to normal function or improved in performance after cervical IA corticosteroid treatment [137]. One limitation of the study was inconsistency in the dosage and type of corticosteroid used, as well as treatment frequency and concurrent therapies provided. Similarly, in clinical practice there are regional and personal differences in the type of corticosteroid used, frequency of treatment and other concurrent therapies. Ultrasound-guided injection of cervical APJ is a well-established technique that is easy to perform [83,84]. However, success is heavily based on operator experience [83]. While the procedure itself requires the use of ultrasound guidance for appropriate administration of the medication, many of the same considerations regarding what medication to use, frequency of administration, and return to work are similar when treating the cervical APJ as for any other IA therapy in a high-motion joint.

#### 9.3.2. Biologic Therapies

Biologic therapies could be considered as an alternative to corticosteroids when the metabolic condition (e.g., insulin resistance) suggests the horse be at an increased risk of laminitis or when medication rules and withholding time would disallow the use of IA corticosteroids. Autologous conditioned serum (ACS) is commonly used to inhibit the effects of IL-1β [138]. In the appendicular skeleton, once-weekly ACS therapy for 3–4 treatments is a commonly used protocol [139]; however, there are no standard treatment schedules for using ACS in cervical APJs, and if treating multiple joints of the cervical spine, this protocol can become cost prohibitive. Anecdotally, the author has seen a positive response in a small number of horses treated with IA ACS in the cervical APJs. In humans with lumbar radiculopathy, ACS and triamcinolone both suppressed pain and disability, but ACS was potentially superior to triamcinolone for long-term pain relief [140]. There is anecdotal evidence to support the use of stem cell therapy for OA in other articular locations. However, to the authors’ knowledge, there are no reports of equine cervical arthropathy cases successfully treated with stem cells at this stage. Further information is warranted before adopting this treatment approach.

### 9.4. Surgical Therapies

#### 9.4.1. Arthroscopy

Arthroscopic evaluation of the APJ may be indicated for diagnostic as well as therapeutic purposes. Surgical considerations could include removal of OC fragments or a better understanding of the cartilage health and integrity. A technique has been described for a lateral approach to the cervical articulation [141]. While the procedure was successfully performed, two of the three horses were euthanized within 96 h of the surgery due to poor prognosis and severe clinical presentation. A more recent report by Tucker et al. describes arthroscopic removal of an OC lesion from C4–C5 in which there was an initial positive response to surgery, but at six weeks post-operatively acute neurologic signs required humane euthanasia [142]. Improvements to the technique and better case selection criteria are necessary before this technique becomes common practice.

#### 9.4.2. Cervical Vertebral Stabilization

While cervical vertebral stabilization has typically been considered only to benefit horses with CVCM and cervical fractures, there is increasing evidence for this procedure to benefit horses with other neck disorders that result in conditions such as neck pain, stiffness, or radiculopathy [143,144]. Indeed, regression of bony arthritic changes was seen 12 months post-operatively with all horses showing resolution of neurologic signs [145]. Two common surgical procedures for cervical stabilization are the use of a Bagby basket [146,147] or a kerf-cut cylinder [148]. A more recent method being applied is with a polyaxial pedicle screw and rod construct [149]. All of these procedures are technically demanding and should be performed only by those with a solid understanding of the cervical anatomy and a high level of surgical training. As imaging modalities continue to improve and evaluation of the cervical spine in multiple planes is possible, we gain a broader understanding of the structures of the cervical spine. For example, altered articular process shape, size and spatial positioning has been shown to result in compression of the spinal cord from the dorsolateral aspect [150]. It is important to continue utilizing our creativity and exploring novel therapeutic options for horses with cervical disease.

## 10. Conclusions and Future Directions

In horses presented for declining performance or behavioral issues, it is of paramount importance to first determine whether the horse may be experiencing pain, and possibly what type of pain. On the surface, this seems like an easy task; however, the diagnosis of cervical pain is not always straightforward, and the clinician must consider all available information: history, observation, static palpation, motion palpation and dynamic evaluation. It is the authors’ opinion that the most critical component moving forward in order to answer this question is appreciating the myofascial examination. The body, and our ability as clinicians to interpret the signs the horse is telling us, must be acknowledged first and foremost. As practitioners, we must develop a systematic approach to the myofascial examination and watch for the subtle signs from the horse. While this seems straight forward, the interpretation of the examination, and fitting it to the clinical picture is complex. We must acknowledge that the reactions are frequently not simply behavioral problems. Diagnostic imaging is indicated to help identify or localize the affected tissue or structures as possible sources of pain, recognizing that all modalities have strengths and limitations, and a clear diagnosis may still not be readily obtained. Looking ahead, the development of imaging modalities capable of evaluation of the cervical region is critical. CT and MRI technology is improving and beginning to allow these examinations to be performed on a more clinical basis. Our understanding of the underlying disease process needs to advance concurrent with our ability to image these areas so that we may interpret the findings and correlate them to the clinical picture. Only at this stage can we begin to have more effective treatments and rehabilitation protocols focused on breaking the pain cycle, improving mobility, and strengthening the cervical spine to support the horse through training. All of these steps must be taken in order to get the horses back to a state of well-being and be able to maintain optimal spinal health over time and through the rigors of their job or athletic endeavors.

## Data Availability

Not applicable.

## Supplementary Materials

The following is available online at https://www.mdpi.com/2076-2615/11/2/422/s1. Video S1: This video highlights a horse that presented for suspect cervical pain and hyperesthesia in the neck region. The horse appears guarded and the thoracolumbar epaxial musculature is hypertonic, though the horse does not openly show pain upon myofascial palpation in that region. The horse does show pain to palpation along the middle gluteal muscle to the caudal sacrum. Mobilization of the cervical spine was not clinically concerning as the horse had moderate ability to laterally bend in both directions. However, the horse shows severe aversion to mobilization of the thoracolumbar region. The horse had minimal radiographic abnormalities of the cervical vertebrae with only mild enlargement at C6–C7. Ultrasonography showed mild osseous irregularity at C6–C7. At post-mortem examination, the gross findings included L5–L6 body ankylosis and L6–S1 dorsal disc protrusion with dural petechial hemorrhage. Histopathology showed severe ganglionitis at the C4–C6 spinal nerve levels and lymphocytic ganglionitis and neuronal loss from T10–T18 and lumbosacral perineural hemorrhage. These complex findings highlight the importance of recognizing that while a horse may show cervical region hyperesthesia, that may in fact not be the primary or only significant region of interest.
